# Functional visual loss: a systematic review and meta-analysis of epidemiology, prognosis and treatment

**DOI:** 10.1038/s41433-026-04648-1

**Published:** 2026-06-26

**Authors:** Neil Ramsay, Helena Tessmann, Justin McKee, Tommaso Ercoli, Jon Stone

**Affiliations:** 1 Deparment of Neurology, NHS Fife, Dunfermline, UK; 2 Neuropsychology Service, South East Scotland Major Trauma Centre, Edinburgh, UK; 3 Princess Alexandra Eye Pavilion and Department of Clinical Neurology, Royal Infirmary of Edinburgh, Edinburgh, UK; 4 Neurology Unit, University Hospital of Sassari, Sassari, Italy; 5 Centre for Clinical Brain Sciences, University of Edinburgh, Edinburgh, UK

**Keywords:** Epidemiology, Risk factors

## Abstract

Functional visual loss is a common issue in neuro-ophthalmology settings. There are many studies focused on diagnosis, but there is limited literature on associated epidemiology, phenomenology, prognosis and treatment. We carried out the first systematic review and meta-analysis assessing these domains. We systematically reviewed PubMed from 1951 for studies reporting clinical data on adults and children with functional visual loss. Data on variables relevant to epidemiology, phenomenology, comorbidity, risk factors, prognosis and treatment were extracted. Random effects meta-analyses were performed where appropriate. Forty-four studies of functional visual loss (*n* = 2284, 67% female) were included. The pooled median age was 43 years (IQR 12.5–43 years). Common associations included facial/ocular trauma (19%, *n* = 288), ocular comorbidity (25%, *n* = 470), headache (39%, *n* = 124) and other functional symptom/disorder (10–29%). Good quality data on psychiatric comorbidity and adverse childhood experience were unavailable. Prognosis data (*n* = 1019, 13 studies) were heterogeneous, but outcome appeared more favourable in paediatric studies. Most studies did not distinguish between symptomatic visual loss and functional visual signs which are asymptomatic (such as spiralled fields) or disproportionate to symptoms (such as severe acuity loss in someone with minor visual symptoms). There are very limited data on treatment. This systematic review highlights some basic epidemiological data and relevant aetiological factors in functional visual loss but also the significant gaps that must be addressed to improve care for this disabling condition.

## Introduction

Functional visual loss, also referred to in the literature as psychogenic or non-organic visual loss, is a cause of vision impairment, especially common in neuro-ophthalmological settings. Functional visual loss falls under the broader category of functional neurological disorder (FND), previously termed “conversion disorder”. FND represents one of the commonest conditions in neurological practice and is typically associated with disability and distress [1, 2]. Modern research positions FND at the interface of neurology and psychiatry as a problem of nervous system functioning rather than structural damage. FND can be understood at a neurological level as a problem with altered predictive processing mediated by brain networks, but also at a psychosocial level where factors such as stress or adverse experience may still be important in many cases [2].

The medical community’s approach to FND has evolved significantly in recent years, with a focus on making a “diagnosis by inclusion”, looking for typical clinical features, instead of exclusion on the basis of normal investigations. In functional visual loss, this usually involves demonstration of variable or “better than reported” acuity using tests such as the fogging test or stereopsis. It does not include patients who are wilfully exaggerating visual loss, who are classified as having factitious disorder or malingering. The management of FND involves integrated multidisciplinary management, rather than purely psychological formulation and treatment [2, 3].

Existing literature on functional visual loss has predominantly focused on the diagnosis with numerous distinguishing tests described for ophthalmologists to demonstrate preserved acuity in the presence of subjective impaired vision[4, 5]. However, there is a lack of synthesised data on the epidemiology, risk factors, comorbidities, prognosis, or management of this condition [6, 7]. In this systematic review, we aim to evaluate this literature to gain a better understanding of functional visual loss.

## Methods

We conducted this systematic review in accordance with the Preferred Reporting Items for Systematic Reviews and Meta-Analyses (PRISMA) guidelines, and we registered our review on the international PROSPERO registry (CRD42025568157). After registration, we added ‘treatment of functional visual loss’ as a search concept and documented this amendment; the search strategy otherwise remained unchanged. We performed an electronic search of PubMed for articles in English and German (owning to authors first language) published between 1951 and 31st October 2024 which used relevant terminology such as “functional visual loss”, “functional blindness”, “medically unexplained visual loss”, “hysterical blindness”, and “non-organic visual loss”, employing free text variants and common word stems. Two authors reviewed the titles and abstracts identified through this search, and the full texts of potentially relevant articles were obtained. These studies were then assessed according to our inclusion and exclusion criteria.

The inclusion criteria were original research articles with clinical data on more than 10 patients diagnosed with functional visual loss, including both adult and paediatric populations. Exclusion criteria were studies focused entirely on other functional visual disorders not related to visual loss or visual field defects (e.g., ocular motility alterations causing diplopia or convergence spasm) and those with less than 10 patients.

Two reviewers independently extracted data and assessed study quality using the Scottish Intercollegiate Guidelines Network (SIGN) criteria for case-control [8] and cohort studies [9] (see supplementary file 1 and 2 for relevant checklists). These criteria have a checklist assessing various aspects of study design including case selection, assessment of confounding factors and statistical analysis. Disagreements were resolved by consensus. The quality of the paper was deemed to be high-quality, acceptable or unacceptable. Those deemed high quality were felt to meet the majority of the criteria in the checklist (with little or no risk of bias). Those felt to be acceptable were felt to meet most criteria with some flaws in study design associated with bias. Those deemed unacceptable were excluded on the basis that either most criteria were not met or that or there were significant flaws relating to key aspects of study design. Examples of features that would deem a paper unacceptable would include: when there isn’t an appropriate or clearly focused question, where there is no mention of confounding factors, when there is no indication of how groups or cases/controls were selected and where outcome measures are not defined.

We recorded demographics (age, sex), epidemiological (population type, any reported incidence or prevalence data), and clinical information (e.g., visual deficit characteristics, monocular vs. binocular loss). We also extracted data on risk factors (e.g., family history, trauma, employment/unemployment, precipitating events), physical and psychiatric comorbidities (e.g., ocular disease, neurological conditions, mood or anxiety disorders), and available follow-up outcomes (whether patients recovered, partially improved, or relapsed). Where sample sizes were not explicitly stated, we derived them from reported percentages or other numeric data within the articles.

Analyses were performed in RStudio (Version 4.4.1, Posit Team, 2024) using the meta package (V7.0.0) [10]. Where studies used comparable methods, we conducted meta-analyses to pool proportions and estimate between-study heterogeneity via a random-effects approach. For studies reporting mean age, we computed an overall weighted mean and median and ranges were noted descriptively when pooling was not possible. We used a random-effects meta-analysis of the female-to-male ratio where study-level data were available, applying the logit transform when necessary. The proportion of monocular vs. binocular loss and colour vision abnormalities were estimated with a random-effects meta-analysis of proportions, with logit or continuity corrections to address boundary values. For studies reporting isolated acuity loss, isolated field loss, or combined deficit, we carried out a multivariate random-effects meta-analysis treating defect type as a moderator. Prognosis was examined in studies with follow-up of at least six months by fitting a multivariate random-effects model to the proportions of full recovery, partial improvement, and relapse, with a 0.5 continuity correction applied to boundary cells. Overall estimates were accompanied by 95% confidence intervals and measures of between-study heterogeneity (I², Q statistic). Risk factors and physical and psychiatric comorbidities were recorded with summary statistics presented descriptively due to the variability in definitions and reporting.

## Results

### Included papers

**Fig. 1:**
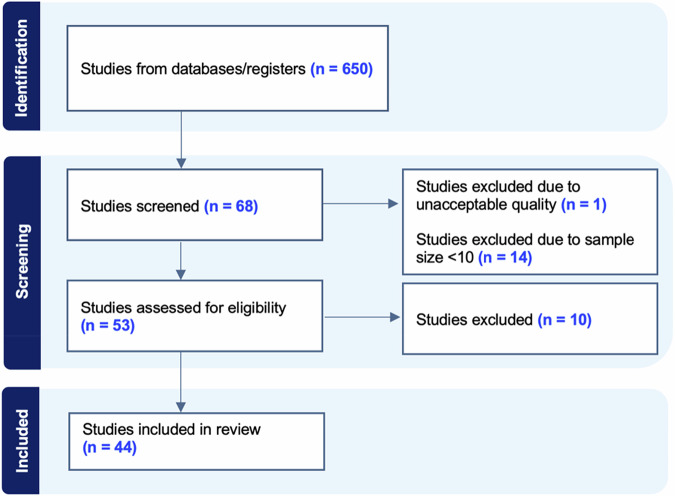
Flow diagram of study selection.

A total of 650 articles were initially identified for screening. After removing duplicates and applying inclusion criteria, 68 potentially relevant papers were identified; of these, 44 met the final criteria for review (Fig. 1) [11–54]. Most papers were case series (*n* = 31) or case-control studies (*n* = 8). Eighteen studies included at least 50 patients, and only 7 enroled 100 or more individuals with functional visual loss. Eight studies were prospective, 34 were retrospective, and 2 had both designs. There were only 2 treatment studies with greater than 10 participants, both of which did not involve controls [26, 40]. Overall, 32 papers were of acceptable quality based on the Scottish Intercollegiate Guidelines Network (SIGN) checklist, with 12 considered high quality 26, 29, 31, 33, 39, 41, 42, 44, 48, 49, 51, 54]; one was excluded for unacceptable quality [55].

### Frequency, age, gender and ethnicity

Reported frequency of functional visual loss varied widely across studies but all suggested a small but reliable presence, especially in neuro-ophthalmology settings. All the studies were in ophthalmology, paediatric or psychiatric clinical settings, and there were no population-based cohorts. A study of 800 ophthalmology outpatients found a frequency of 5.3% [56], while a further study estimated that functional visual loss accounted for 5% of an ophthalmologist’s caseload, though both lacked clear timeframes, so it is difficult to draw conclusions [15]. In one of the few studies that included a prospective cohort, 70 patients were recruited from two specialist neuro-ophthalmology clinics over a 12-month period [51]. A study in a specialist neuro-ophthalmology service reported an average of 10.2 cases per year over seven years [11]. A study of 109 cases of non-organic visual loss over 5.5 years in a German university hospital, equated to 19.8 cases per year [52]. A larger study reported an annual incidence of 3.5% for “medically unexplained visual loss” in children presenting to a specialist paediatric eye casualty clinic, based on 85 cases from 2397 new attendances [29].

**Fig. 2:**
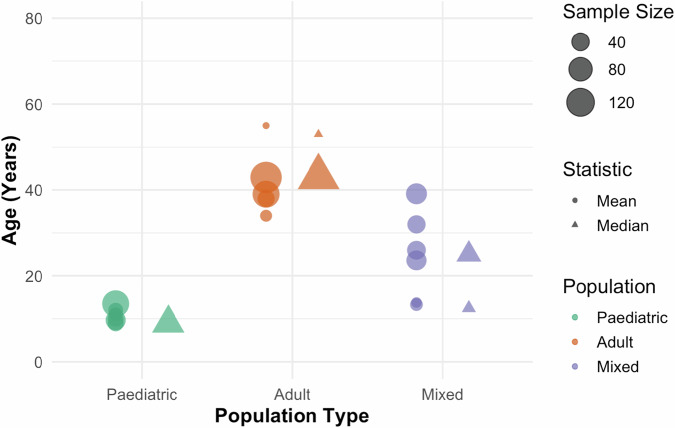
Mean and median ages across population groups. Sizes of circles or triangles represent number of subjects in the study.

Forty-one studies, including 2284 participants, reported sex distribution and 28 reported age at time of clinic review. Seventeen studies focused on paediatric cohorts (904 participants), 16 investigated mixed populations (847 participants), and 10 were adult-only (517 participants); one paper did not specify [48]. Insufficient studies specifically reported age of onset to synthesise that data. Often the age was presented variably, and data includes both age of onset and age at clinic review. Nineteen studies provided a mean age, six gave a median age, and 21 recorded minimum and maximum ages. The youngest reported age was 3.5 years, and the oldest was 89 years (IQR: 30.5 years). Reported ages varied by study type and tended to cluster into two groups, with paediatric cohorts reporting median or mean ages between 8 and 14, and adult cohorts clustering around 35–45 years. Mixed-age studies showed broader variation. The most common age range across studies fell between 10 and 40 years (see Fig. 2).

The pooled proportion of female participants was 67.2% (95% CI: 63.2–71%), suggesting females were overrepresented but substantial heterogeneity across studies (I^²^ = 73.7%, *p* < 0.0001) limits interpretability.

A meta-analysis suggests slightly higher proportions of female participants in paediatric (70%, 95% CI: 65.8–73.8%) and mixed-age (67.9%, 95% CI: 63.4–72.1%) compared to adult-only populations (61.5%, 95% CI: 46–75%; Q = 1.5, *p* = 0.5) but these differences were not statistically significant. Only a limited number of studies provided data on patient ethnicity, which were insufficient to draw reliable conclusions regarding the overall ethnic distribution of functional visual loss.

### Phenomenology

Ten studies, with a total of 267 participants, systematically described the type of visual defects which we categorised as visual acuity only, visual field only, and both visual field and visual acuity defects, but their methodological approaches were similar enough that their data could be combined into a meta-analysis. Thirty-three studies had a more varied approach to the collection of phenomenological data, which limited our ability to complete a meta-analysis. Although some of these papers were focused on other topics, such as comorbidity [39], electrophysiology [46] and assessing different diagnostic tests [48] most provided limited ophthalmological phenomenology. Very few papers comment on the symptoms and experience of the patients themselves, and most presented objective data (i.e. acuity, visual evoked potentials, field perimetry) without making it clear whether the patient was complaining of visual loss or not.

A study of 109 individuals with functional visual loss described concomitant ophthalmological symptoms in 41(38%) patients with 23(21%) having blurred or distorted vision, 11(10%) reporting reduced visual acuity in the dark, 5% stated they had double vision with 2% describing photopsia [52]. This study also presented field abnormalities as either being concentric (*n* = 62), diffuse (*n* = 10) or nasal (*n* = 2). A study of 30 paediatric patients did not separately describe the type of visual field defect but again showed a majority of patients (80%) with binocular visual acuity loss [37].

A study of 85 children again showed a predominance of bilateral visual acuity loss 72/85 (84.5%), concentric narrowing and atypical scotomas were detected in 49/85 (58%) [54]. A study of 58 patients also reported bilateral symptoms in the majority (*n* = 41, 71%) [44] with 34/58 (60%) having severely impaired visual acuity ( ≤ 20/40) [44]. Among the 28 patients with visual field issues, 9 patients described concentric fields, and all but one of these patients had variable patterns of visual field testing on sequential assessment [44].

A study of 133 patients again described associated visual field defects with constricted visual fields in 52/133 (39%), spiral in 30/133 (23%) [41]. Convergence spasm was described commonly 29/133 (22%), whereas ptosis and blepharospasm were infrequent, occurring in 3% (4/133) and 2% (2/133) of patients, respectively, in line with other studies [47].

A study which mainly employed the term “hysterical amblyopia” assessed 19 patients aged 9–50 [47]. The majority 15/19 (79%) had bilateral visual loss, with 3 patients (16%) having severe visual loss including “tubular visual” field and variability on repeat visual assessments, similar to recent studies [44].

A multivariate random-effects meta-analysis on the 10 studies which systematically described the type of visual defects, found a 57.5% frequency of combined field and acuity deficits (95% CI: 36.9–77.9), 30.7% for isolated acuity (95% CI: 9.3–52.1), and 11.8% for isolated field (95% CI: –1.7 to 25.4). Pairwise contrasts indicated that combined deficits were significantly more common than isolated field deficits (z = 3.3, *p* = 0.003), but no other differences were statistically significant. Importantly, these estimates reflect clinician-rated impairment, based on formal visual testing (e.g., acuity charts, perimetry), rather than self-reported symptoms of visual loss. All studies included in the analysis required some observable or testable visual dysfunction to confirm the diagnosis, but many papers did not specify whether all patients with observable deficits actually had visual symptoms.

### Monocular versus binocular presentation

**Fig. 3:**
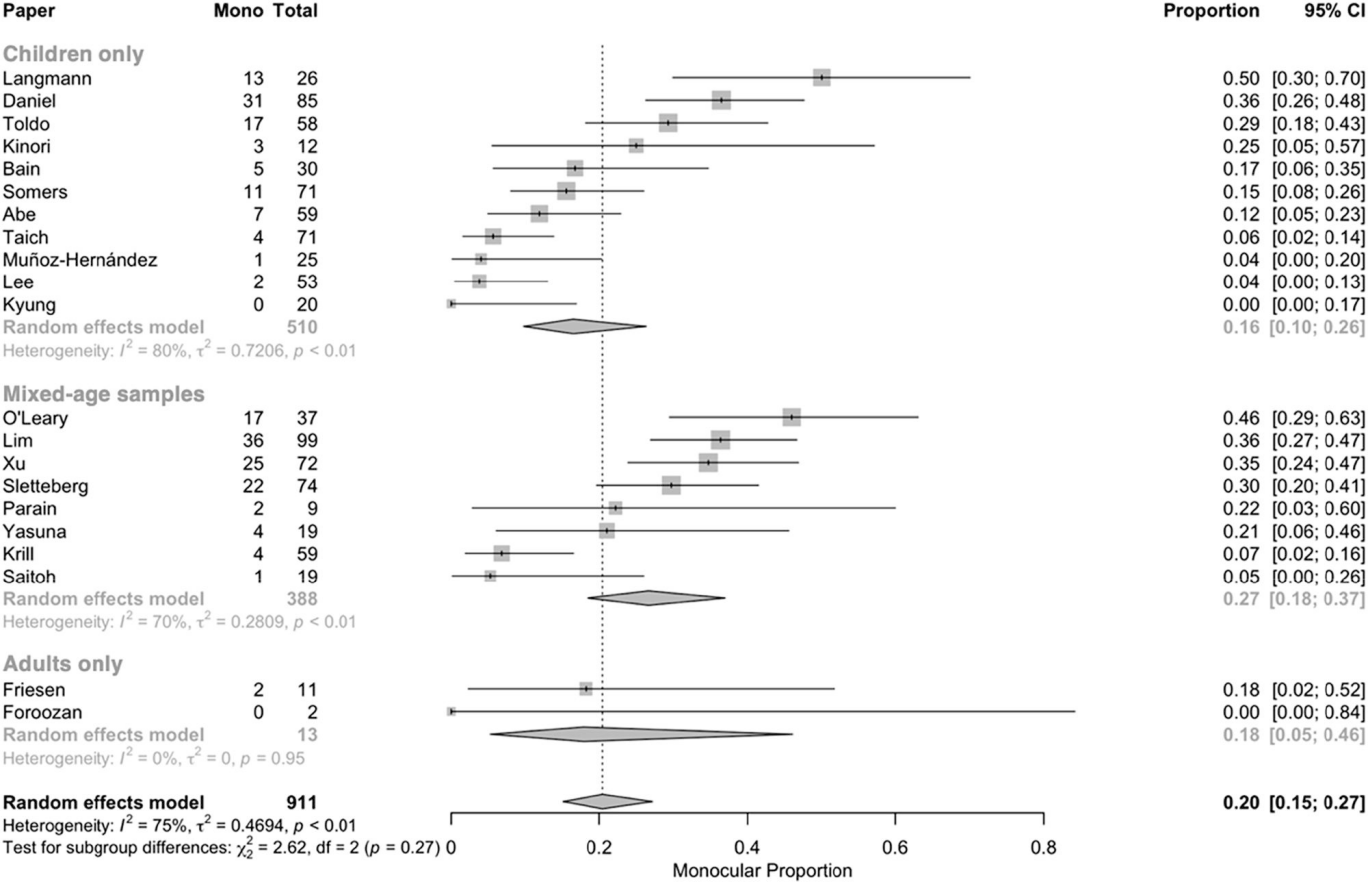
Forest plot of the proportion of monocular visual loss across different population Subgroups (Paediatric, Adult, Mixed).

A separate meta-analysis was conducted on 21 studies (911 participants) that reported whether functional visual loss was monocular or binocular in presentation. The random-effects model estimated that 20% (95% CI: 14–27%; I² = 74%, *p* < 0.01) of cases were monocular, suggesting that binocular presentations are more common ( ~ 80%). Subgroup analysis by age group yielded proportions of 16% (paediatric), 18% (adult), and 27% (mixed), without a significant difference across groups (*p* = 0.3). The analysis showed substantial heterogeneity (I² = 74%, *p* < 0.01) in the reported proportions of monocular visual loss. Detailed subgroup analysis results are provided in Fig. 3.

### Colour vision

Seven studies included in this review commented on changes in colour vision [13, 21, 22, 30, 34, 54, 57]. The prevalence of colour vision deficits varied across different studies and populations. Overall, a total of 55 cases out of 285 patients (19.2%) experienced some form of colour vision abnormality on testing. The majority of studies simply reported impaired colour vision on Ishihara chart testing. Some reported inconsistencies between Ishihara red-green testing and a testing with a Nagel anomaloscope [13]. No studies provided a qualitative description of the colour vision impairments that patients experienced. It is therefore unclear if this is an “incidental” finding on standard ophthalmic assessment and to what extent people with functional visual loss do experience disturbances of colour vision.

### Physical symptom/disorder comorbidity

Fifteen studies, including 890 patients with a combination of paediatric, adult and mixed cohorts, including data on comorbidity of physical symptoms/disorders alongside a functional visual loss diagnosis. The data was too heterogeneous to allow a formal meta-analysis, so is reported descriptively below.

### Functional symptoms and disorders other than vision

Five studies described the presence of other functional symptoms and disorders alongside functional visual loss, varying from 10 to 29% of patients in their respective cohorts [12, 15, 26, 38, 51]. A study of 100 adults found that 18 (18%) had another functional symptom, including seven with functional seizures and two with functional limb weakness, two with chronic abdominal pain and two with unexplained hearing loss [51]. In a study of 59 children, eight had concurrent functional hearing loss, albeit how this was assessed is not described [26]. A study of 17 adults focused on functional visual loss in idiopathic intracranial hypertension (IIH) [38]. Five of these 17 patients (29%) were noted to have positive features of FND on their neurological examination (i.e. give way weakness/non-localising sensory and motor findings). In a case series of 10 adults, one patient had concurrent functional seizures [58]. A further study of 42 patients, including a mixed adult/paediatric cohort noted 10/42 (24%) had a somatisation disorder (without further characterisation) [15].

At least eight studies detailed information on other probable functional symptoms. Two papers detailed the comorbid presence of abdominal pain 24% and 37%, respectively [44, 51]. A small number of patients were noted to have dizziness in two papers, 7% [52] and 2% [51]. “Chronic pain” was also noted by two studies to be present in 20% [51] and 7% (limb pain) of subjects [44].

In summary, the frequency of other FND symptoms in patients with functional visual loss is much higher than in the population, but data on other symptoms is too sparse to draw any firm conclusions.

### Ocular comorbidity

Overall, the frequency of other ocular conditions was reported in seven studies, including 116 patients out of a total of 470 (25%), ranging from 6% (*n* = 6/109) [52] to 62% (*n* = 62/100) [51]. In a study of 59 patients, six patients (10%) had concurrent ocular pathology, which in three cases didn’t objectively cause visual acuity/field loss, while the other three patients had a diagnosis of macular degeneration, optic atrophy and optic neuritis, respectively [13]. A paediatric treatment study noted 7 out of 59 patients (12%) presented with concurrent eye disease or pain, with no further detail given. In a further study were 6 patients out of 109 (6%) had “eye pain” [52]. In a cohort of 17 patients with IIH with functional visual loss 4 patients (24%) had abnormal disc pallor or gliosis, but it was felt that the severity of visual impairment wasn’t compatible with the ophthalmoscopy findings [38]. A study of a mixed cohort found 45 patients out of 84 (53%) had concurrent ocular pathology, the most common of which was strabismus and “other motility” disorders 17, 38%), 67 (15%) had other “organic problems”, and 6 (13%) had retinal abnormalities [21]. A study showed 9 patients out of 42 (21%) had associated ocular pathology, including posterior vitreous detachment, exotropia, congenital amblyopia (2 patients), punctate keratitis, cataracts and band keratopathy [15]. In conclusion, either comorbid or precipitating ocular pathology appears to be a strong risk factor for the development of functional visual loss.

### Physical or ocular trauma

Physical trauma, both generally and specifically (ocular and facial), was also a clear theme as a precipitating factor for the development of functional visual loss.

Six studies highlighted either recent or previous facial, head or ocular trauma, including 56 out of 288 patients (19%) [13, 25, 26, 33, 41, 51].

In a study, 6/59 (10%) patients had ocular trauma as an immediate precipitant to their functional visual loss. Two of these patients developed binocular symptoms despite having trauma to one eye [13]. A study which specifically focused on functional visual loss in a cohort of Cambodian refugees found that 9/16 (56%) had suffered an injury to the eye or face. In this cohort, the visual symptoms appeared to develop slowly, even up to years after the initial injury [25]. In another study, 12/59 (20%) paediatric patients had minor facial or ocular injuries that were not substantial enough to damage ocular function but immediately preceded visual loss [26].

Nine studies with a total of 674 patients found that 101 (15%) had some form of physical trauma described non-specifically or elsewhere in the body that was associated with the onset of their functional visual loss [12, 13, 17, 36, 39, 41, 42, 51, 53]. Three studies highlighted cases where the onset of symptoms occurred after a surgical procedure, including 22 out of a total 305 (7%) [30, 33, 41], with one study reporting functional visual loss in two patients after cataract surgery [30].

This literature mirrors similar themes to those of the ocular comorbidity literature. Facial or ocular trauma appears relevant as a predisposing or precipitating factor for functional visual loss in some cases.

### Other neurological conditions (including headache/migraine)

The frequency of other neurological conditions alongside a diagnosis of functional visual loss, in cohorts *not* selecting for specific comorbidities, e.g. IIH, was reported in nine studies ranging from 2% (*n* = 1/59) to 53% (*n* = 31/58) with a total of 227 patients out of 700 (32%) [13, 15, 33, 36, 41, 44, 49, 51, 52].

Headache or migraine was the most commonly reported comorbidity and was specifically was reported in 6 studies (2 studies did not differentiate headache disorders from other neurological diagnoses), ranging from 14% (*n* = 6/42) to 42% (*n* = 56/133) with a total of 124 patients out of 319 (39%) [15, 33, 36, 41, 51, 52]. Two studies gave a more detailed breakdown of the headache presentation [36, 51]. Migraine was specifically mentioned present in 17% (*n* = 24/138) to 18% (*n* = 18/100) [36, 51]. Smaller numbers of patients—6% (*n* = 8/138) and 2% (*n* = 2/100) had a diagnosis of trigeminal neuralgia [36, 51].

Epilepsy was specifically reported in 3 studies ranging which were 6% (*n* = 6/100), 5% (*n* = 2/42) and 4% (*n* = 5/133), respectively [15, 41, 51].

One study focused on a cohort of 17 patients with both functional visual loss and a diagnosis of IIH [38, 59]. Interestingly, 13 of the 17 patients had no evidence of disc swelling. Three patients required management with topiramate for headache symptoms in the absence of disc swelling, which may raise questions about comorbid migraine although it is not explicitly mentioned.

The literature on neurological comorbidity, including headache, supports this as a common comorbid condition as well as a likely precipitating and perpetuating factor in functional visual loss.

### Psychiatric comorbidity

Eighteen studies commented on psychiatric comorbidity, but with variable reporting of psychiatric diagnoses; therefore, the data are presented descriptively. Furthermore, it was evident that the prevalence of various mental health diagnoses was lower even than the expected frequency in the population, calling into question the reliability of the data.

Only 3 of these 18 studies involved a prospective cohort [23, 29, 45]. Only two studies referred to diagnostic criteria, i.e. DSM-IV studies [15, 42], three studies collected information either through some sort of interview [13, 23, 45] or a brief review of “psychological history” [29] one study asked about psychological factors in a “non-direct” way [43]. Two studies with the highest frequency of psychiatric disorders, 30% and 50%, respectively, collected information more systematically through patient-reported questionnaires [36, 51]. One mixed retrospective and prospective study systemically collected data of mental health comorbidity(linked with primary care records) 42% (n = 42/100) were found to have a diagnosis of depression, 13% were found to have a diagnosis of anxiety (all had concurrent depression), 7% had PTSD and only 2% had a diagnosis of a personality disorder [51]. Many studies collected information on psychiatric history through case note review but did not reference methodology for collecting or coding [23, 33, 39, 53, 54]. The remaining studies had no clear methodology for collecting information on psychiatric comorbidity.

Psychiatric comorbidities were recorded as follows; Depression (18%, 10 studies, 106/604 patients), Anxiety (10%, 5 studies, 44/452 patients), Bipolar disorder (3%, 5 studies 10/293), Personality disorder (11%, 5 studies, 26/235), Post-traumatic stress disorder (5%, 3 studies 14/259), Attention-deficit/hyperactivity disorder (ADHD) (8%, 4 studies, 16/198 patients), Substance misuse (10%, 4 studies 17/176 patients), Eating disorders (3%, 4 studies 7/222 patients) and Psychosis/schizophrenia (3%, 4 studies 5/176).

Although many studies report psychiatric comorbidity, the lack of systematic prospective assessment suggests that this data is not reliable.

### Potential aetiological risk factors

The current paradigm of understanding the aetiology of FND with predisposing, precipitating, and perpetuating factors was less distinct within the functional visual loss literature. There was rarely a distinction made in any of the studies between predisposing remote factors (such as adverse childhood experience) and precipitating factors (such as eye injury, migraine or a very recent stressor) [2]. These were often conflated in studies, particularly if they fell within a broad category of “psychosocial stressors”. Eight studies, including a total of 367 patients, highlighted “accidents” as a common precipitant for functional visual loss involving 150 (41%) patients. This term was used in such a broad way (everything from physical, trauma, car accidents to wider psychosocial stressors) that it was not possible to amalgamate data, so we describe individual studies below.

### Psychosocial factors

Similar methodological issues emerged with the study of psychosocial factors, which were rarely studied in a systematic or controlled way.

Three studies involving a total of 7 out of 247 patients (3%) highlighted a history of abuse in childhood (either sexual, physical or neglect) [20, 36, 54]. Of note, the majority of studies appeared to rely on self-reporting of abuse or neglect during interview rather than a systematic enquiry regarding a history of abuse or neglect.

Sixteen studies, including a total of 849 patients, commented on 233 patients (27%) who had a psychosocial factor of some kind (i.e. parental relationship breakdown, housing issues etc.) that were presumed to be involved in predisposing or precipitating the patient’s functional visual loss symptoms [13, 17, 20, 21, 24, 36–39, 41–45, 50, 51]. One prospective study reported that 17% (*n* = 17/100) of patients had a psychosocial factor related to their symptoms, compared to none of 36 healthy controls or 21 other causes of visual loss [51].

Several studies provided very limited information on these psychosocial factors, with descriptions including “familial circumstances”, “social/psychosocial stressors” [20, 36, 37, 39]. This was broken down slightly more in other studies [42–45, 51] referencing psychosocial stressors such as: “stress at work,”, “sibling rivalry/jealousy”, “general stress,” and “around the time of marriage.” A further study described “emotional crises” as the being a trigger factor in 5/59 patients (8%) [13].

Psychosocial stressors were an especially clear theme in paediatric literature, for example, “school stress”, “home stress”, “divorce”, “bullying” and “loss of a parent” [54]. In a study of 25 children [19],10 (40%) had a “home conflict”, 9 (36%) had a “school conflict”, and 5 (25%) had a parental divorce either occurring or pending [21]. Another study recorded that children with functional visual loss (*n* = 129) were significantly more likely to have a mother who did not work outside the home compared to 145 controls (71% vs 29%) [50]. Six studies involving 57 out of 254 children (22%) highlighted “school issues” as being a precipitating factor development of functional visual loss such as “poor academic achievement”, “school conflict” or bullying [15, 20, 21, 24, 45, 51].

Five studies, including 330 patients (4 of which involved paediatric populations) concluded that 34 (10%) of these patients had a “motivation” of wanting glasses considered relevant to the development of functional visual loss [20, 36, 37, 43, 54]. No information was provided on how that judgement was made. There was an inference inferred that the desire or glasses was to receive “further attention”. One study did not draw a line between functional visual loss and malingering in this context [35].

A single uncontrolled study reported that in 56/84 cases (67%) there was some financial incentive, but this included disability benefits and workers’ compensation which may be appropriate when visual impairment is genuinely experienced [21].

In summary, psychosocial factors were a commonly reported factor related to functional visual loss, especially paediatric patients. However, there was little controlled data, and such stressors are common in the general population.

### Prognosis

Sixteen studies discussed prognosis in patients with functional visual loss (see Table 1). Of these, 13 studies comprising 1019 patients included follow-up periods longer than six months.

**Table 1:** Prognostic data and study-level characteristics of 16 studies of functional visual loss.

Author (Year)	*N*	Follow-up Duration	Follow-up Rate (%)	Population Type	Mean Age	Design	% Female	Worse (%)	Same (%)	Improved (%)	Complete Remission (%)
**Visual and Symptoms Assessment**											
Abe (2000)	59	2–5 years post-treatment	85	Paediatrics	8–9 years	P	73	14	–	86	–
Toldo (2010)	58	Mean 7.4 months	96	Paediatrics	10	R	67	–	7	–	93
Suppiej (2010)	49	Mean 57 months	91	Paediatrics	9.3	R	63	–	–	–	92
Catalano (1986)	23	1–67 months (mean 34)	96	Paediatrics	11	R	70	–	–	–	96
Daniel (2017)	85	Median 1.2 months	67	Paediatrics	Median 9	P	63	–	–	14	25
Rada (1973)	20	1–3 years	90	Paediatrics	Not specified (range 7–18)	P	–	–	10	55	–
Sletteberg (1989)	54	2 months-24 years	54	Mixed	24	R	72	–	55	–	45
Xu (2001)	72	1 day-45 months	52	Mixed	32	P	64	–	8	83	–
Kathol (1983)	42	Mean 53 months	100	Mixed	32	R	79	–	55	19	26
**Visual Assessment Only**											
Kinori (2011)	12	2–108 months	100	Paediatrics	10	R	75	–	8	17	75
Friesen (1966)	11	6–31 years	100	Adult	55	R	18	27	27	27	–
Lim (2005)	138	31.5 months	26	Mixed	Not specified	R	74	–	–	–	58
Barris (1992)	45	Mean of 114 days	62	Mixed	26	R	80	–	22	36	42
**Follow-up Period Not Specified or Very Short**											
Somers (2016)	85	2 weeks	96	Paediatrics	11	R	74	–	–	–	88
Lee (2016)	53	Not specified	100	Paediatrics	10	R	62	–	4	96	–
Taich (2004)	71	Not specified	88	Paediatrics	9	R	66	–	–	80	8

R Retrospective, P Prospective. Outcome columns reflect symptom change at follow-up.

The longer follow-up studies varied in duration from nine months to 32 years [32] and were grouped dichotomously into those where follow-up included some formal symptom assessment (9 studies) [15, 19, 20, 24, 26, 29, 42, 44, 46], or those that used objective visual assessments such as acuity or visual fields, which may not have corresponded with visual symptoms (4 studies) [11, 32, 33, 36].

Three studies used a combination of objective clinical parameters to assess improvement (i.e. acuity and perimetry) alongside a patient-rated questionnaire to assess improvement [19, 26, 46].

Three studies presented objective measurements (i.e. visual acuity and perimetry) alongside patient-reported symptom non-standardised outcome [20, 29, 42, 44]. One paper simply reported the proportion of patients with a return to “normal vision” [20].

One paper presented improvement in visual fields and acuity, alongside psychiatric assessment to assess improvement [26], and in a further paper, acuity and field improvement were presented alongside a clinician-rated outcome of improvement [15]. A further four studies simply provided clinician-rated acuity and field improvement with no patient-reported outcomes or other metrics [11, 32, 33, 36].

An attempt was made to complete a meta-analysis of the prognostic studies categorised by those with and without an assessment of visual symptoms. However, the statistical measure of heterogeneity was so high (QE = 88,684, *p* < 0.0001) that result of the random effects model was felt to be too unreliable.

Of the nine studies that included an objective assessment of acuity and fields alongside some form of symptom assessment, six involved a purely paediatric population and three were mixed. Six of these studies reported whether there was “complete remission” in symptoms. The range rate among all studies was wide 25–96% but of note, the frequency in three paediatric only studies was far higher at 92–96% [20, 42, 44]. Five studies reported whether patient’s symptoms improved on follow-up, providing a broad range of recovery rates from 14 to 86% [15, 24, 26, 29, 46]. Unlike in the studies that assessed complete resolution of symptoms, there was no relationship to whether the sample was paediatric or adult.

Despite considerable heterogeneity, there are some signals that paediatric populations do have higher rates of recovery compared to mixed populations.

### Treatment

Three of the authors of this paper reviewed practical strategies for treatment of functional visual loss noting the lack of evidence [7]. Some literature relevant to treatment in children and adolescents has also been summarised in a bibliometric mapping study [60], although this mainly looked at what diagnostic labels and treatments were used with no synthesis of outcomes.

Within the literature, we found two cohort treatment studies in functional visual loss of 10 or more patients [26, 40]. This involved 33 children with an approach focused on using suggestion and encouragement to support improvement in visual function. Children had different numbers of sessions (mean 3, range 1–7). 28 (88%) recovered within 4 sessions. There was only follow-up data in for 14 patients, 12 of whom had maintained their visual acuity on follow-up (which varied from 1 to 4 years).

Another treatment study reported on the use of two session transcranial magnetic stimulation to the occipital cortex for 10 patients with functional visual loss, embedded in a report on similar treatment for various functional symptoms [40]. In this study six patients had ‘immediate complete recovery’ and three a “dramatic improvement”. Two had a relapse of their symptoms with subsequent improvement. There’s no record of systematic follow up of the other eight patients.

Several other studies comment on different treatment approaches without systematic data. Two studies emphasised the importance of explanation and reassurance to all patients [15, 22]. One study highlighted the importance of screening for psychiatric comorbidity and referred 28 out of 85 (25%) to child and adolescent psychiatry [54]. Several studies highlighted the use of placebo treatments (albeit they are not labelled as such) to assess if they benefited patients, including 10 out of 42 patients who benefited from eyedrops [15]. In one study, one out of three patients, was recorded as having ‘improved symptoms’ from weak glasses or contact lenses [20]. An editorial commented that a proposed treatment trial of young people with functional visual loss turned out to be non-viable due to low recruitment [60].

## Discussion

This systematic review is the first in the literature to look at the demography, phenomenology, risk factors, comorbidity, prognosis and treatment of patients with functional visual loss. A striking theme from the literature is the large number of publications focused on how to diagnose functional visual loss, with little information presented on the actual symptomatology and the patients’ experience of their symptoms. Few studies included patient-reported measures that assessed the functional impact of symptoms, such as whether visual loss affected patients’ ability to perform activities of daily living. We found it especially difficult to determine what proportion of patients in these studies had asymptomatic functional visual findings on assessment (e.g. a spiralled fields in the context of no subjective symptoms of a visual field abnormality) or disproportionate functional visual findings (e.g. severe acuity deficit in someone complaining or mild or intermittent visual blurring) as opposed to impactful and persistent functional visual loss. In functional disorders doctors often think about patients with symptoms but little to find on examination. In the area of visual symptoms, the opposite often occurs and does not appear to have been adequately recognised in the literature.

Functional visual loss is often diagnosed appropriately in ophthalmology settings but is less often referred on to other specialties such as psychological services [7]. A recent survey of 119 UK ophthalmologists confirmed that the majority felt they had little training in or confidence in how to manage functional visual loss [61]. This is reflected in the content of the literature, particularly the low number and small size of treatment studies for patients with functional visual loss.

The systematic review allowed for a limited assessment of frequency, age of onset and sex in functional visual loss. There are clear gaps in the literature, particularly around prevalence and incidence, with studies only reporting proportions from their own selected clinic population and nothing within a wider population context.

Most patients across the dataset fell between the ages of 10 and 40. Importantly, these figures reflect age at the time of assessment—not age of symptom onset—a distinction highlighted in prior literature where functional symptoms in adults may have begun years earlier [62]. A female predominance (67%) was observed, consistent with other FND subtypes and functional disorders in general. This pattern is broadly in line with data from functional movement disorder cohorts, where women account for approximately 70% of cases, although female predominance may be slightly less marked in younger patients. In contrast, studies of functional seizures have shown an earlier peak in onset, particularly among women, suggesting that age and gender distributions may vary across FND subtypes. However, the apparent differences in paediatric representation could partly reflect reporting or referral bias across cohorts [62]. Although we can state that binocular visual loss is more common (80%) the literature does little to understand the patients experience of their symptoms, or why some patients develop unilateral versus bilateral symptoms.

There was evidence of an excess of ophthalmic comorbidity as well as ocular and facial trauma alongside functional visual loss. This would fit current paradigms in FND, in which the type of physical comorbidity often acts as a precipitating factor which determines the nature of the FND subtype. Examples include patients with epilepsy who develop functional seizures, or those that have an acute vertiginous illness such as benign positional paroxysmal vertigo who then develop persistent functional dizziness /persistent postural positional dizziness [2]. No studies formally assessed the impact of managing ocular comorbidity on functional visual loss. This is clearly another area of importance for multidisciplinary treatment studies in the future. We know that in many subtypes of FND, treating related comorbidity can lead to improvement in FND symptoms. For example, in patients with frequent migraine with ongoing functional limb weakness, treating the underlying migraine could improve symptoms in some patients [63].

Five studies reported the presence of other functional somatic symptoms (10–29%) alongside functional visual loss, including a much higher frequency of other FND symptoms (10-20%) such as limb weakness, seizures and deafness than the general population level of FND symptoms (around 0.2%) [1]. We do not have data to understand how often functional visual loss occurs as a milder symptom alongside more debilitating FND symptoms, for example, seizures or weakness. Our clinical experience suggests that it’s a more common minor complaint, especially ipsilateral to functional limb weakness and sensory loss. For example, in one study of 107 individuals with functional limb weakness, 38 had symptoms of visual disturbance [64].

It is also our clinical experience that migraine and photophobia are common predisposing, precipitating and perpetuating risk factors for functional visual loss. The role of migraine as a precipitating factor is illustrated in a case report from our group, in which the severe photophobia a patient experienced after a migraine persisted and developed into severe bilateral functional visual loss [65]. Although no larger studies within the literature formulated this relationship, several studies highlighted (39% over the 6 studies) the presence of headache, most likely predominantly migraine, with functional visual loss. This likely represents an underestimate, but the high proportion clearly indicates the importance of screening for and treating headache (particularly migraine) in functional visual loss, especially given that we have a range of potential treatments for this condition. A wider literature on the interaction between migraine and FND indicates some potential commonalities in mechanism and aetiology [63].

The literature on psychiatric comorbidity was either retrospective and notes-based or relied on non-systematic prospective assessment with interview or standard questionnaire [36, 51]. We think it’s difficult to draw conclusions about the prevalence of psychiatric comorbidity in functional visual loss as a consequence and our conclusion is that properly conducted prospective studies using standardised measures are needed.

Much of the literature mentions various psychosocial factors related to functional visual loss presentations. This was a particular theme in paediatric studies, particularly around relevant school or home stressors. Clearly, these factors will be relevant to many of these cases, given our understanding of the role of psychosocial stressors in FND [66]. However, the lack of control groups within the literature and the ubiquitous nature of stress, make it difficult to draw firm conclusions. It’s a difficult area to study generally in FND. Psychosocial risks do appear clearly relevant for some individuals, but it’s an issue that is highly prone to confirmation bias [66]. The same is true for the concept of ‘secondary gain’ which is very hard to study and for which there is no clear evidence from the studies included, or indeed the wider literature on FND [3].

The literature did have substantial data on prognosis in functional visual loss, with data from at least 16 studies, but only nine involved some assessment of symptom improvement. Symptoms resolution rates were broad 14–96%, but much better in paediatric cohorts 93–96%. This is higher than other subtypes of FND such as seizures or motor symptoms. This would correlate with anecdotal experience of improvement, especially in children, although we have many adults with persistent symptoms in our own clinics [67]. As most studies likely included patients with mild symptoms or patients with ocular findings and not symptoms (that would be assumed could easily resolve on follow-up) it again means significant caution must be required when interpreting this prognostic data.

This review assessed a diverse range of studies over 70 years in a number of clinical settings. As expected, this included a broad range of terminology such as visual hysteria and medically unexplained visual loss. Earlier studies in the majority involved more granular description of individual cases with most (though not all) of the larger cohort studies being in the last 30 years. Although terminology around functional visual loss has changed the concept in of itself has not in terms of a description of an individual reporting visual loss who can be demonstrated through testing to have normal or much better vision. What was more evident in earlier studies where the differences in social and medical professional attitudes around the role of mental health and stress as the “cause” of visual loss particularly as the Freudian or “conversion” dominated at this time. Our impression reading this material is that epidemiological data from older studies remains of relevance but need to be interpreted within their historical context.

This review has several limitations. The majority of studies were single-cohort, single-centre uncontrolled retrospective data with obvious methodological issues that those entail. The literature also included several historical studies with consequential impact on terminology, technical assessments and socio-cultural factors. Our literature only included papers in English and German owing to the authors’ native languages. Moreover, while we compared paediatric-only and mixed populations, a mixture of different cohort types and the relatively small number of studies led to wide confidence intervals and limited power. These considerations highlight the need for caution when interpreting the subgroup findings.

In summary, this first systematic review of the demography, phenotype, comorbidity and prognosis of functional visual loss provides a useful first step to understand of this patient group. We propose a number of key research learning points (Box 1) to help improve our understanding of functional visual and to develop much-needed treatments for this patient group.

Box 1 A road map for future studies of the epidemiology and treatment of functional visual loss

- Studies should delineate a diagnosis of functional visual loss on the basis of impactful visual symptoms, and not just findings on visual assessment.
- Research should prioritise consistent diagnostic criteria, clear outcome definitions, and both clinician- and patient-rated measures.
- Prospective assessment of comorbidities, especially their time course and related psychosocial factors to better understand how functional visual loss develops.
- Larger prospective treatment studies, with well described treatments are needed to understand outcome and the effect of proposed treatments.

## Supplementary information

Supplementary File 1: SIGN- checklist-for-cohort-studies

Supplementary File 2: SIGN-checklist-for-case-control-studies

Supplementary details.

## Data Availability

The datasets generated during and/or analysed during the current study are available from the corresponding author on reasonable request.
