# Supplementary material for: Functional visual loss: a systematic review and meta-analysis of epidemiology, prognosis and treatment
Source: Eye (Lond). 2026 Jun 26;40(12):1784–93. doi: 10.1038/s41433-026-04648-1 (PMC13463093; doi:10.1038/s41433-026-04648-1)
Supplement: Supplementary file 1 [file 41433_2026_4648_MOESM3_ESM.docx]

**Supplementary File 1 – SIGN Checklist for Cohort Studies**

**Supplementary File 2 – SIGN Checklist for Case Control Studies**
