# Supplementary material for: Functional visual loss: a systematic review and meta-analysis of epidemiology, prognosis and treatment
Source: Eye (Lond). 2026 Jun 26;40(12):1784–93. doi: 10.1038/s41433-026-04648-1 (PMC13463093; doi:10.1038/s41433-026-04648-1)
Supplement: Supplementary file 3 [file 41433_2026_4648_MOESM2_ESM.rtf]

	Methodology Checklist 4: Case-control studies	
Study identification (Include author, title, year of publication, journal title, pages)
	
Guideline topic:	Key Question No:	Reviewer:	
Before completing this checklist, consider:
1.	Is the paper really a case-control study? If in doubt, check the study design algorithm available from SIGN and make sure you have the correct checklist.
2.	Is the paper relevant to key question? Analyse using PICO (Patient or Population Intervention Comparison Outcome). IF NO REJECT (give reason below). IF YES complete the checklist.	
Reason for rejection: Reason for rejection: 1. Paper not relevant to key question □   2. Other reason □ (please specify):	
Section 1:  Internal validity	
In an well conducted case control study:	Does this study do it?	
1.1	The study addresses an appropriate and clearly focused question. Unless a clear and well defined question is specified in the report of the review, it will be difficult to assess how well it has met its objectives or how relevant it is to the question you are trying to answer on the basis of the conclusions.
	Yes  
Can't say 	No 
	
Selection of subjects	
1.2	The cases and controls are taken from comparable populations. Study participants may be selected from the target population (all individuals to which the results of the study could be applied), the source population (a defined subset of the target population from which participants are selected), or from a pool of eligible subjects (a clearly defined and counted group selected from the source population. If the study does not include clear definitions of the source population it should be rejected.
	Yes  
Can't say 	No 
	
1.3	The same exclusion criteria are used for both cases and controls. All selection and exclusion criteria should be applied equally to cases and controls. Failure to do so may introduce a significant degree of bias into the results of the study.
	Yes  
Can't say 	No 
	
1.4	What percentage of each group (cases and controls) participated in the study? Differences between the eligible population and the participants are important, as they may influence the validity of the study. A participation rate can be calculated by dividing the number of study participants by the number of eligible subjects. It is more useful if calculated separately for cases and controls. If the participation rate is low, or there is a large difference between the two groups, the study results may well be invalid due to differences between participants and non-participants. In these circumstances, the study should be downgraded, and rejected if the differences are very large.
	Cases:
Controls:	
1.5	Comparison is made between participants and non-participants to establish their similarities or differences. Even if participation rates are comparable and acceptable, it is still possible that the participants selected to act as cases or controls may differ from other members of the source population in some significant way. A well conducted case-control study will look at samples of the non-participants among the source population to ensure that the participants are a truly representative sample.
	Yes  
Can't say 	No 
	
1.6	Cases are clearly defined and differentiated from controls. The method of selection of cases is of critical importance to the validity of the study. Investigators have to be certain that cases are truly cases, but must balance this with the need to ensure that the cases admitted into the study are representative of the eligible population. The issues involved in case selection are complex, and should ideally be evaluated by someone with a good understanding of the design of case-control studies. If the study does not comment on how cases were selected, it is probably safest to reject it as a source of evidence.
	Yes  
Can't say 	No 
	
1.7	It is clearly established that controls are non-cases. Just as it is important to be sure that cases are true cases, it is important to be sure that controls do not have the outcome under investigation. Control subjects should be chosen so that information on exposure status can be obtained or assessed in a similar way to that used for the selection of cases. If the methods of control selection are not described, the study should be rejected. If different methods of selection are used for cases and controls the study should be evaluated by someone with a good understanding of the design of case-control studies.
	Yes  
Can't say 	No 
	
ASSESSMENT	
1.8	Measures will have been taken to prevent knowledge of primary exposure influencing case ascertainment. If there is a possibility that case ascertainment can be influenced by knowledge of exposure status, assessment of any association is likely to be biased. A well conducted study should take this into account in the design of the study.

	Yes  
Can't say 	No 
Does not apply 	
1.9	Exposure status is measured in a standard, valid and reliable way. The primary outcome measures used should be clearly stated in the study. If the outcome measures are not stated, or the study bases its main conclusions on secondary outcomes, the study should be rejected. Where outcome measures require any degree of subjectivity, some evidence should be provided that the measures used are reliable and have been validated prior to their use in the study.
	Yes  
Can't say 	No 
	
CONFOUNDING	
1.10	The main potential confounders are identified and taken into account in the design and analysis. Confounding is the distortion of a link between exposure and outcome by another factor that is associated with both exposure and outcome. The possible presence of confounding factors is one of the principal reasons why observational studies are not more highly rated as a source of evidence. The study should indicate which potential confounders have been considered, and how they have been allowed for in the analysis. Clinical judgement should be applied to consider whether all likely confounders have been considered. If the measures used to address confounding are considered inadequate, the study should be downgraded or rejected. A study that does not address the possibility of confounding should be rejected.
	Yes  
Can't say 	No 
	
STATISTICAL ANALYSIS	
1.11	Confidence intervals are provided. Confidence limits are the preferred method for indicating the precision of statistical results, and can be used to differentiate between an inconclusive study and a study that shows no effect. Studies that report a single value with no assessment of precision should be treated with extreme caution.
	Yes  
	No 
	
Section 2:   OVERALL ASSESSMENT OF THE STUDY	
2.1	How well was the study done to minimise the risk of bias or confounding?  Rate the overall methodological quality of the study, using the following as a guide: High quality (++): Majority of criteria met. Little or no risk of bias.  Results unlikely to be changed by further research. Acceptable (+): Most criteria met. Some flaws in the study with an associated risk of bias, Conclusions may change in the light of further studies. Low quality (0): Either most criteria not met, or significant flaws relating to key aspects of study design. Conclusions likely to change in the light of further studies.
	High quality (++) □
Acceptable (+) □
Unacceptable – reject 0 □	
2.2	Taking into account clinical considerations, your evaluation of the methodology used, and the statistical power of the study, do you think there is clear evidence of an association between exposure and outcome?	Yes  
Can't say 	No 
	
2.3	Are the results of this study directly applicable to the patient group targeted by this guideline?	Yes  	No 	
2.4	Notes. Summarise the authors conclusions. Add any comments on your own assessment of the study, and the extent to which it answers your question and mention any areas of uncertainty raised above..
